# Association of clinical and radiographic findings with the outcomes of 93 patients with COVID-19 in Wuhan, China

**DOI:** 10.7150/thno.46569

**Published:** 2020-05-15

**Authors:** Lingli Li, Lian Yang, Shan Gui, Feng Pan, Tianhe Ye, Bo Liang, Yu Hu, Chuansheng Zheng

**Affiliations:** 1 Department of Radiology, Union Hospital, Tongji Medical College, Huazhong University of Science and Technology, Wuhan 430022, China; 2 Hubei Province Key Laboratory of Molecular Imaging, Wuhan 430022, China; 3 Institute of Hematology, Union Hospital, Tongji Medical College, Huazhong University of Science and Technology, Wuhan, Hubei 430022, China

**Keywords:** X-Ray Computed Tomography, Viral Pneumonia, COVID-19, Outcome

## Abstract

**Rationale**: To retrospectively analyze serial chest CT and clinical features in patients with coronavirus disease 2019 (COVID-19) for the assessment of temporal changes and to investigate how the changes differ in survivors and nonsurvivors.

**Methods**: The consecutive records of 93 patients with confirmed COVID-19 who were admitted to Wuhan Union Hospital from January 10, 2020, to February 22, 2020, were retrospectively reviewed. A series of chest CT findings and clinical data were collected and analyzed. The serial chest CT scans were scored on a semiquantitative basis according to the extent of pulmonary abnormalities. Chest CT scores in different periods (0 - 5 days, 6 - 10 days, 11 - 15 days, 16 - 20 days, and > 20 days) since symptom onset were compared between survivors and nonsurvivors, and the temporal trend of the radiographic-clinical features was analyzed.

**Results**: The final cohort consisted of 93 patients: 68 survivors and 25 nonsurvivors. Nonsurvivors were significantly older than survivors. For both survivors and nonsurvivors, the chest CT scores were not different in the first period (0 - 5 days) but diverged afterwards. The mortality rate of COVID-19 monotonously increased with chest CT scores, which positively correlated with the neutrophil-to-lymphocyte ratio, neutrophil percentage, D-dimer level, lactate dehydrogenase level and erythrocyte sedimentation rate, while negatively correlated with the lymphocyte percentage and lymphocyte count.

**Conclusions**: Chest CT scores correlate well with risk factors for mortality over periods, thus they may be used as a prognostic indicator in COVID-19. While higher chest CT scores are associated with a higher mortality rate, CT images taken at least 6 days since symptom onset may contain more prognostic information than images taken at an earlier period.

## Introduction

In December 2019, a cluster of patients with acute respiratory disease recognized as coronavirus disease 2019 (COVID-19) was identified in Wuhan, Hubei Province, China 1, 2. Initially, most patients had an exposure history that could be traced to the Huanan Seafood Wholesale market directly or indirectly, while increasing evidence has eventually indicated human-human transmission within families or in hospitals 3-7. The infection has rapidly spread from Wuhan to other areas worldwide likely through air travel 4, 5, 8, which has raised a public health emergency of international concern. By April 1, 2020, 750,890 confirmed cases have been detected worldwide, with a mortality rate as high as 4.8% 9.

The clinical characteristics and radiographic manifestations of COVID-19 patients have been recently discovered 1, 10-14. Most patients have mild disease, with common symptoms of fever, cough and myalgia or fatigue at onset 1. However, fatal outcomes including acute respiratory distress syndrome (ARDS) and multiorgan dysfunction, have occurred in severe patients 11. Chest CT plays an important role in the diagnosis and monitoring of COVID-19 15-18. Previous studies showed a typical time course of CT demonstrations of COVID-19 aggravation, including subpleural ground glass opacity (GGO) in the early stage, enlarged GGO with a crazy-paving pattern and partial consolidation in the progressive stage, and subsequent extensive consolidation in the bilateral lungs 14, 19-23. Fatal hypoxemia can occur as pulmonary involvement progresses 1, 14, but the degree of pulmonary involvement that indicates fatal outcome remains unclear. To date, several previous studies 24-29 have revealed different clinical and imaging features between survivors and nonsurvivors or between non-ICU patients and ICU patients, and some potential risk factors for mortality of patients with COVID-19 have been preliminarily discovered, such as older age, neutrophil-to-lymphocyte ratio (NLR), lymphocyte count and D-dimer level. These risk factors have mainly been identified with logistic regression models, Cox proportional hazard ratio models, or Kaplan-Meier methods, which miss the dynamic information of clinical and imaging features during follow-up. However, the dynamic profile of clinical features in patients with COVID-19, including lymphocyte count, neutrophil count, D-dimer level, and Troponin T level, may reflect the severity of this disease 12, 24. Furthermore, it remains unclear whether there is a correlation between the dynamic profiles of radiographic and clinical features in COVID-19.

This study analyzes the temporal changes of radiographic-clinical features in survivors and nonsurvivors, which is hypothesized to be of prognostic value for clinical decisions.

### Methods

The Ethics Committee of Union Hospital, Tongji Medical College, Huazhong University of Science and Technology (No. 2020-0026) approved this retrospective study. The requirement for informed consent was waived. This study was conducted in accordance with the Declaration of Helsinki.

### Patients

From January 10, 2020, to February 22, 2020, a cohort of 93 consecutive patients admitted to this single center were enrolled in the study. The inclusion criteria were as follows: (1) patients diagnosed with COVID-19 by a positive nucleic acid test according to published standard protocols from the National Health Commission of the People's Republic of China 30; (2) patients who underwent a series of chest CT scans (at least 2 times), with laboratory tests on the same day as chest CT; and (3) patients who either died or were discharged before February 22, 2020. The exclusion criteria were patients with non-COVID-19-related death events (e.g., malignant diseases and acute coronary syndromes). The treatment and discharge criteria for patients with COVID-19 were in reference to the published standard protocols from the National Health Commission of the People's Republic of China 30. The serial chest CT examinations, clinical features, onset symptoms and signs, treatments and outcome events of all the patients were recorded. The clinical features and outcomes were monitored up to February 22, 2020, by which date all the patients either died or were discharged, hence resulting in a survivor group and a nonsurvivor group. The flow chart of patient recruitment was depicted in the supplemental data (Figure S1).

### Clinical features

The clinical features included complete blood count, liver and renal function, lactate dehydrogenase (LDH), creatine kinase, coagulation profile, and myocardial enzymes. The NLR was also calculated. For each patient, the serial clinical features (including the clinical features of patients with COVID-19 on admission and the corresponding clinical features on the same days as the chest CT scans were performed) were retrieved from the electronic medical records.

### Chest CT protocol

All patients underwent chest CT with two commercial multidetector CT scanners (Philips Ingenuity Core128, Philips Medical Systems, Best, the Netherlands; SOMATOM Definition AS, Siemens Healthineers, Germany) covering the apex to the base of the lung. The CT scan parameters were as follows: a tube voltage of 120 kVp with automatic tube current modulation, a matrix size of 512 × 512, a thickness of 1.5 mm and an increment of 1.5 mm, with either hybrid iterative reconstruction (iDose level 5, Philips Medical Systems, Netherlands) or a pulmonary B70F kernel and a mediastinal B30f kernel (Siemens Healthineers, Germany).

### Image interpretation

Chest CT images were retrieved from the picture archiving and communication system (Carestream, Canada) for image feature scoring. Chest CT findings in patients with COVID-19 were defined using internationally standard nomenclature 31-33. The typical CT findings for COVID-19 included GGO, crazy-paving pattern, consolidation and mixed pattern 14, 19, 20, 34, which were defined in the supplementary data. The distribution of these abnormal findings was also noted as predominantly subpleural, random, or diffuse 35. The extent of pulmonary abnormalities on thin-slice CT was evaluated by a conventional semiquantitative scoring system 35. Each lung was divided into three regions: superior (above the carina), middle (the carina up to the inferior pulmonary vein), and inferior (below the inferior pulmonary vein). Each lung region (a total of 6 lung regions, bilaterally) was scored according to the following indicators: score 0, 0% involvement; score 1, less than 25%; score 2, 25% to 50%; score 3, 50% to less than 75%; and score 4, 75% or more. The sum of all scores provided an overall picture of lung involvement (the maximum CT score including both lungs was 24). The frequency of each demonstration was compared between the two groups in 5 periods (0 - 5 days, 6 - 10 days, 11 - 15 days, 16 - 20 days, and > 20 days since symptom onset). The chest CT demonstrations and scores were assessed by three radiologists (CSZ, BL, and LY, who had 26, 25 and 22 years of experience in thoracic radiology, respectively) using both axial CT images and multiplanar reconstruction images, with the decisions reached by consensus.

### Statistical analysis

All the statistical analyses were performed using IBM SPSS Statistics Software (version 24; IBM, New York, USA). Five periods were defined based on the time since symptom onset: 0 - 5 days, 6 - 10 days, 11 - 15 days, 16 - 20 days, and > 20 days. First, the chest CT scores and clinical features of the survivors and nonsurvivors were compared based on the variable types and their distribution. Second, linear mixed models with repeated measures were developed to assess the radiographic progression profile and the clinical features progression profile between the survivors and nonsurvivors. Third, the binary multivariable logistic regression model was developed for outcomes, the binary dependent variables (nonsurvivors were coded as 1, and survivors were coded as 0), with the independent variables of chest CT scores and time since symptom onset. Fourth, descriptive graphic analysis was used to examine the relationship between the chest CT score and the clinical features within a longitudinal view. The Kendall coefficient was computed to measure the strength of the correlations between the chest CT scores and clinical features. A two-sided α of less than 0.05 was considered statistically significant.

## Results

### Basic characteristics

The final cohort consisted of 93 patients with confirmed COVID-19, with a mean age of 51.0 ± 17.5 years, and 41 (44%) patients were male. A total of 32 (34%) patients had comorbidities, including diabetes (11 [12%]), hypertension (5 [5%]), cardiovascular disease (4 [4%]), chronic obstructive pulmonary disease (8 [9%]), and malignancy (4 [4%]) (Table 1). Fever (89 [96%]), dry cough (66 [71%]), and fatigue (63 [68%]) were the most common initial symptoms (Table 1).

For clinical outcomes, 68 (73%) patients were regarded as survivors and were discharged, and 25 (27%) patients were considered fatal, with a mortality rate of 27%. Among the 68 survivors, there were 42 (62%) females and 26 (38%) males. Among the 25 nonsurvivors, there were 10 (40%) females and 15 (60%) males. Compared to survivors, nonsurvivors were significantly older (69.0 ± 10.5 vs 43.7 ± 13.1 years, p < 0.01) and were more likely to have dyspnea and comorbidities (20 [80%] vs 12 [18%], 16 [64%] vs 12 [18%] patients; p < 0.01 for both). The duration (the time from symptom onset to different outcomes) was not significantly different between survivors and nonsurvivors (p = 0.735) (Table 1). A total of 89 (96%) patients received intravenous immunoglobin (Table 1). The treatments were significantly different between nonsurvivors and survivors (p < 0.01 for all) (Table 1). Compared to survivors, for nonsurvivors, the NLR and lymphocyte percentage were significantly decreased on admission, while the LDH, erythrocyte sedimentation rate (ESR) and C-reactive protein (CRP) levels were significantly increased (Table 2).

### Chest CT demonstrations

**Figure 1:**
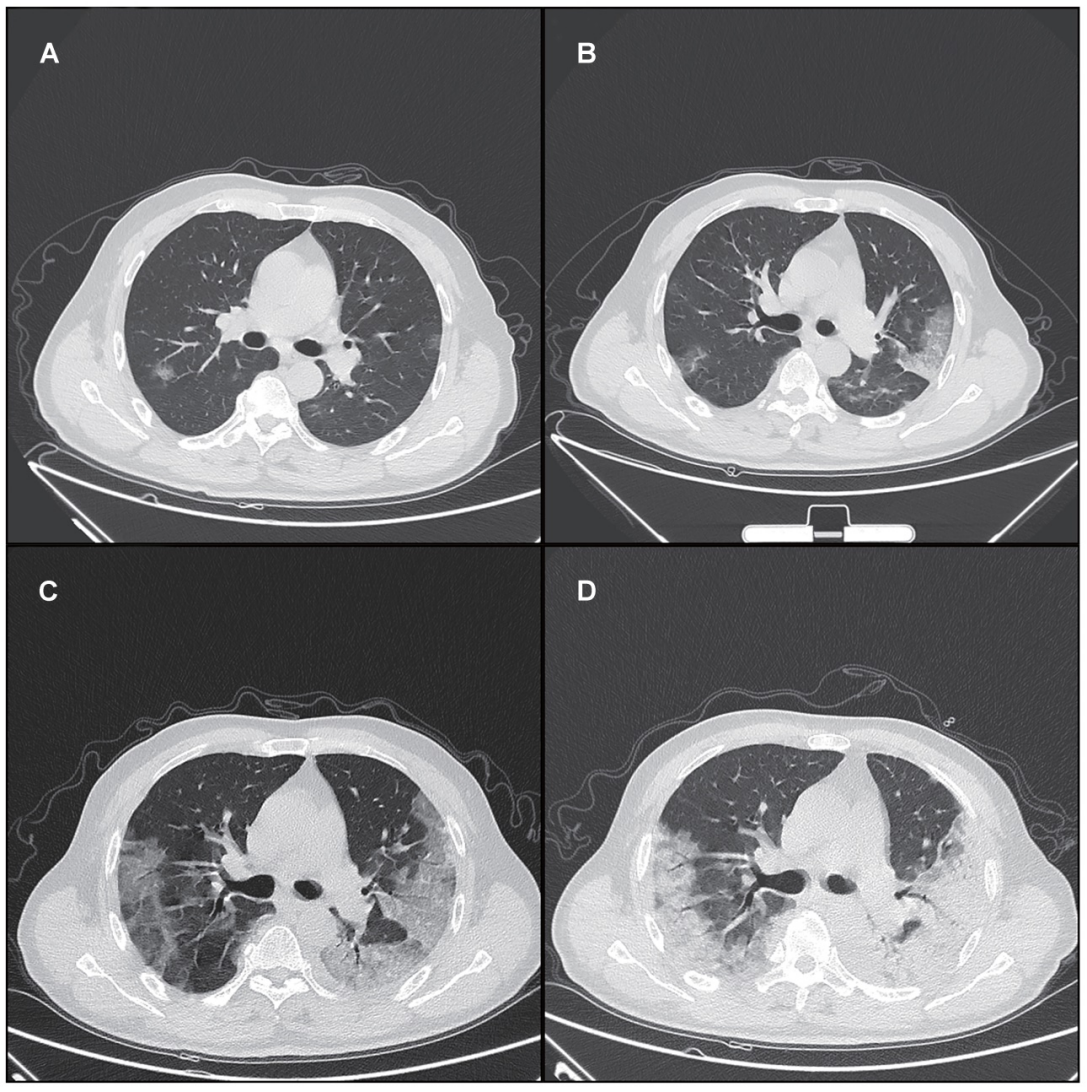
Typical series of chest CT demonstrations in a 65-year-old male patient with COVID-19 in different periods. (A) In the first period (0 - 5 days), GGO; (B) In the second period (6 - 10 days), a crazy-paving pattern (GGO with superimposed inter- and intralobular septal thickening); (C) In the third period (11 - 15 days), a mixed pattern (GGO, crazy-paving pattern and consolidation); (D) In the fourth period (16 - 20 days), a mixed pattern (crazy-paving pattern and consolidation). All images have the same window level of - 600 and window width of 1600. GGO: ground glass opacity.

A total of 175 chest CT scans were scored in this study. A total of 140 (80%) chest CT scans demonstrated bilateral infiltrates, and 31 (18%) chest CT scans showed unilateral infiltrates, whereas 4 (2%) chest CT scans had no abnormal findings. Pulmonary involvement identified from chest CT (including bilateral infiltrates, unilateral infiltrates, and no involvement) was not significantly different between survivors and nonsurvivors (p > 0.05 for all) (Supplementary Table S1). The main chest CT demonstrations of COVID-19 included GGO, a crazy-paving pattern, consolidation and a mixed pattern (Figure 1). The frequency of each demonstration was compared between survivors and nonsurvivors in 5 periods as specified in Supplementary Table S2. A crazy-paving pattern in the third period (11 - 15 days) was significantly more common in nonsurvivors than in survivors (p < 0.05) (Supplementary Table S2). The crazy-paving pattern disappeared in the last period (> 20 days) for all patients.

### Progression of chest CT scores and clinical features

**Figure 2:**
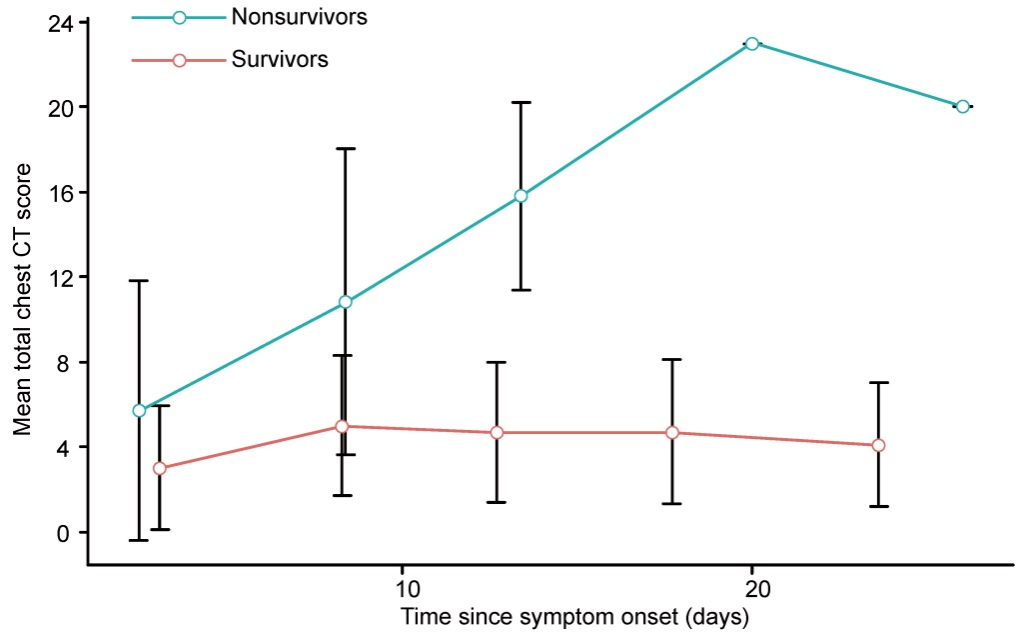
Graph shows the mean chest CT scores plotted against the mean time (from symptom onset to chest CT scan) in different periods for patients with COVID-19. Error bars indicates the standard deviation.

The chest CT scores for each clinical outcome group were compared for different periods. Within the nonsurvivors, the chest CT scores generally increased in the first 4 periods (up to 20 days) and then slightly decreased in the last period (> 20 days). In contrast, the chest CT scores in the survivors generally increased during the first 2 periods (up to 10 days), remained stable during the next period (11 - 15 days), slightly increased during the 4th period (16 - 20 days), and then decreased in the last period (> 20 days). For both survivors and nonsurvivors, the chest CT scores were not significantly different in the first period (0 - 5 days, p = 0.412), but the differences became significant in the next 4 periods (from 6 days onward, p < 0.01 for all) (Supplementary Table S3, Figure 2). The number of days from symptom onset to each chest CT scan in each period was not significantly different between survivors and nonsurvivors (p > 0.05 for all, Supplementary Table S3).

Linear mixed models with repeated measures were developed for the serial chest CT scores and clinical features with clinical outcomes. The results showed that the measurements of the serial chest CT scores and some of clinical features were significantly different between the survivors and nonsurvivors, and the changes in these measurements over periods were also significantly different between the survivors and nonsurvivors (Supplementary Table S4). In addition, some measurements (including both CT scores and clinical features) were found to change significantly over periods, except for the change in lymphocyte percentage, lymphocyte count and neutrophil percentage (Supplementary Table S4).

### Risk of death

The mortality rate was closely associated with chest CT score for all periods with a monotonic increasing trend (p < 0.01), as indicated by the binary multivariable logistic regression for clinical outcome and time since symptom onset (Table 3). Noticeably, the same chest CT scores could have conferred a different mortality rate in different periods.

### Analysis of radiographic and clinical features

**Figure 3:**
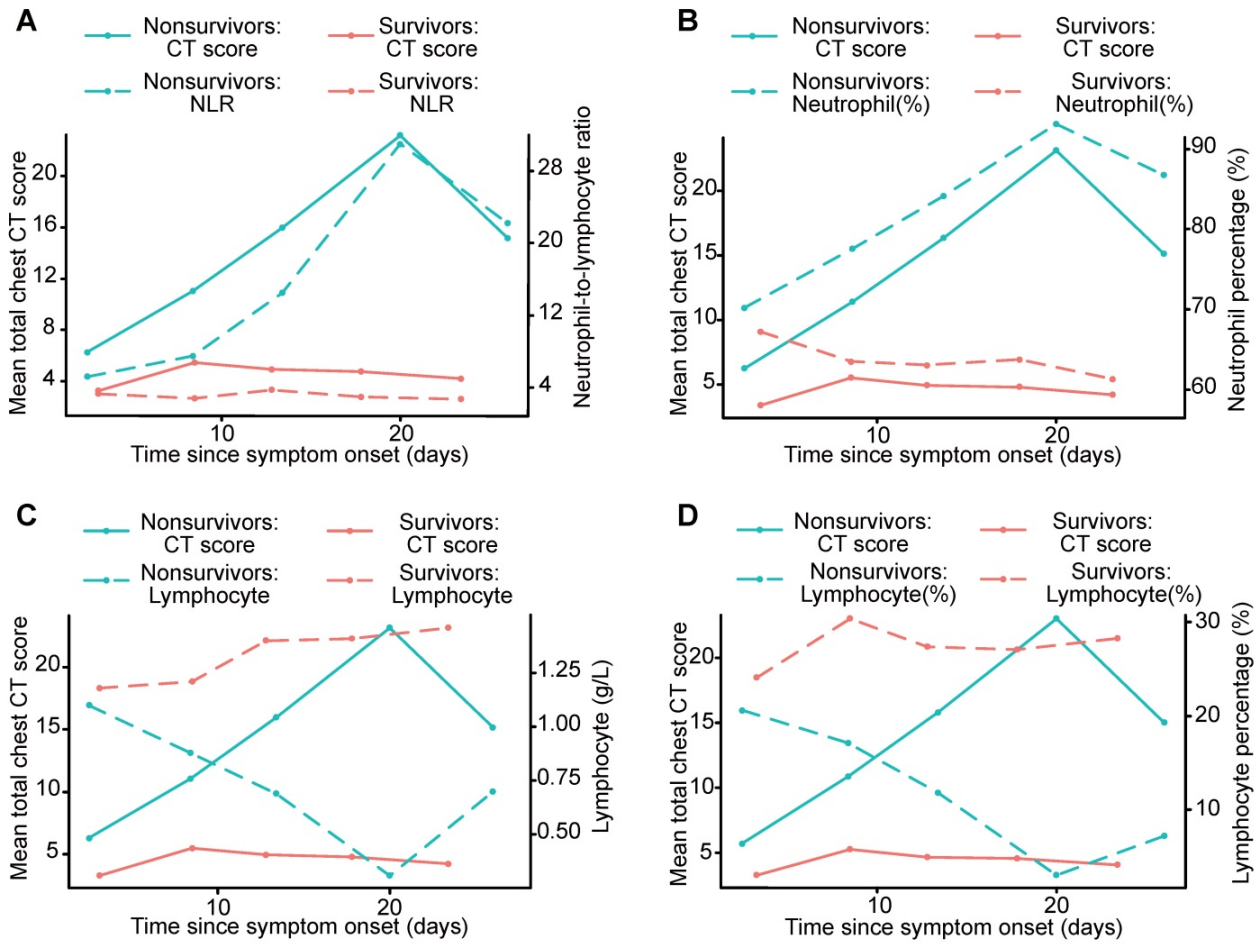
Graph shows the mean chest CT scores and the NLR, neutrophil percentage, lymphocyte count and lymphocyte percentage plotted for different periods, including survivors and nonsurvivors. The curves for mean chest CT scores showed temporal trends that were consistent with those of the NLR and neutrophil percentage, and divergent points between the outcome groups were present in the second period (A, B). The curves for mean chest CT scores showed temporal trends that were opposite those of the lymphocyte count and lymphocyte percentage (C, D). NLR: neutrophil-to-lymphocyte ratio.

Serial chest CT scores were well correlated with D-dimer, LDH, and ESR levels (Kendall coefficient, 0.445, 0.477 and 0.473, p < 0.01, respectively) and moderately correlated with the NLR, lymphocyte percentage, lymphocyte count, and neutrophil percentage (Kendall coefficient, 0.228, - 0.218, - 0.294 and 0.264, p < 0.01, respectively), as demonstrated in Table 4 and Figure 3.

## Discussion

This study presents a retrospective analysis of serial chest CT and clinical features in patients with COVID-19 for assessing temporal changes and investigating how the changes differ in survivors and nonsurvivors. The results revealed that chest CT scores were not different in the first period but diverged afterwards between the survivors and nonsurvivors. The mortality rate of COVID-19 monotonously increased with chest CT scores, and CT images taken at least 6 days after symptom onset may contain more prognostic information than images taken at an earlier period. Serial chest CT scores positively correlated with the NLR, neutrophil percentage, and D-dimer, LDH and ESR levels, while negatively correlated with the lymphocyte percentage and lymphocyte count during the course of the disease.

Our results revealed that the average age of nonsurvivors with COVID-19 was older than that of survivors, with more dyspnea and comorbidities. Previous studies have also indicated that older patients with comorbidities are at higher risk of infection and fatal respiratory complications 11, 28. Dyspnea was one of the most common symptoms in nonsurvivors and was more likely to progress to ARDS. Severe disease onset might result in death due to massive alveolar damage and progressive respiratory failure 1, 6. We found a higher mortality rate of 27% than that recently reported 1, 10, 11, 36, probably because of the difference in sample sizes and patient inclusion criteria.

COVID-19 infection can cause severe lower respiratory tract infection with bilateral, basal and peripheral of lung injury 37. In our study, the main CT demonstrations in different periods included GGO, a crazy-paving pattern, consolidation and a mixed pattern, in agreement with previous studies 14, 19-21, 34. In survivors, lung abnormalities on chest CT peaked in the second period (6 - 10 days) after initial symptom onset, with mainly consolidation and a higher total CT score, and gradually resolved thereafter with decreasing total CT scores and diminishing crazy-paving pattern. The crazy-paving pattern in the third period (11 - 15 days) was significantly more common in nonsurvivors than in survivors (p < 0.05). The crazy-paving pattern tended to decrease or even disappear in the last period (> 20 days) in survivors. In contrast, in nonsurvivors, consolidation and mixed patterns were consistently observed in the final 2 periods (> 16 days) with more extensive lung involvement and slower resolution, implying the progression of lung injury. These findings suggested consolidation as an indicator of disease progression and potentially an alert for patient management, which has also been suggested previously with a much smaller cohort of patients 21.

For the survivors and nonsurvivors, the chest CT scores were not significantly different in the first period (0 - 5 days) but diverged afterwards, implying a non-prognostic role of CT scores in the first period. In survivors, the chest CT scores generally increased in the first 2 periods (up to 10 days), remained stable during the next period (11 - 15 days), slightly increased during the 4th period (16 - 20 days), and then decreased in the last period (> 20 days), similar to a previous report 20. In nonsurvivors, the chest CT scores generally increased in the first 4 periods (up to 20 days), while they slightly decreased during the last period, which was probably due to the insufficient sample size. These findings may suggest that chest CT, if performed at least 6 days after symptom onset, is a prognostic indicator for COVID-19.

Serial chest CT scores positively correlated with the NLR, neutrophil percentage, and D-dimer, LDH and ESR levels, while negatively correlated with the lymphocyte percentage and lymphocyte count during the course of the disease. Compared to survivors, in nonsurvivors, more severe lymphopenia developed over time, which could be a critical factor associated with disease progression and mortality to provide evidence for the pathological findings of interstitial mononuclear inflammatory infiltrates in both lungs in COVID-19 38. Lymphocyte subsets and the NLR were helpful in the early screening of critically ill patients with COVID-19 25. Higher NLR and neutrophil percentage were observed over time in nonsurvivors, likely due to neutrophilia's role in the cytokine storm induced by virus invasion 12. The NLR is also used for assessing the severity of virus infection and systemic inflammation, and an elevated NLR is considered to indicate poor clinical prognosis in acute-on-chronic hepatitis B liver failure and high-grade serous ovarian cancer 39, 40. Higher D-dimer, LDH and ESR levels were measured over time in nonsurvivors in our study, ESR and LDH levels are traditionally used as indicators of systemic inflammation 39, and D-dimer reflects fibrin breakdown after fibrinolytic system activation. Elevated D-dimer level has been detected in patients with disseminated intravascular coagulation 41, and coagulation activation may be related to sustained inflammatory response 12, which may be associated with the pathologic mechanisms in the death of patients with COVID-19. D-dimer level greater than 1 µg/L can indicate poor prognosis in patients with COVID-19 at an early stage 26. Lower lymphocyte percentage and lymphocyte count, and higher NLR, neutrophil percentage, D-dimer, LDH and ESR levels may predict a worse prognosis in patients with COVID-19. Thus, chest CT scores may also reflect the severity of COVID-19 as well as the clinical features, which can provide important information in clinical practice.

The mortality rate was closely associated with chest CT scores with a monotonously increasing trend. In addition, the same chest CT scores could have conferred a different mortality rate in different periods, and higher scores in the earlier phase of the disease indicated a worse prognosis, as suggested by our results. This study revealed a feasible semiquantitative tool as a prognostic indicator for COVID-19.

The main limitations of the present study are the retrospective nature of the design and the consequent incomplete CT follow-up data in severe patients. When patients with COVID-19 develop severe respiratory distress and hypoxemia, chest CT examination is more difficult in clinical practice, which was the main cause of sample deficiency for the last 2 periods (> 15 days) in nonsurvivors.

## Conclusions

Chest CT scores correlate well with the risk factors for mortality over periods, thus they may be used as a prognostic indicator in COVID-19. While higher chest CT scores are associated with a higher mortality rate, CT images taken at least 6 days since symptom onset may contain more prognostic information than images taken at an earlier period.

## Figures and Tables

**Table 1:** Demographics and baseline characteristics of patients with COVID-19

	All patients (n=93)	Survivors (n=68)	Nonsurvivors (n=25)	p value
**Demographics**				
Age, years	51.0±17.5	43.7±13.1	69.0±10.5	**<0.01^1^**
Sex				
Female	52(56%)	42(62%)	10(40%)	0.098^2^
Male	41(44%)	26(38%)	15(60%)	
**Signs and symptoms**				
Fever	89(96%)	66(97%)	25(100%)	1.0^2^
Dry cough	66(71%)	59(87%)	8(32%)	<0.01^2^
Fatigue	63(68%)	45(66%)	20(80%)	0.38^2^
Expectoration	29(31%)	17(25%)	12(48%)	<0.05^2^
Diarrhea	16(17%)	12(18%)	4(16%)	1.0^3^
Anorexia	37(40%)	21(31%)	17(68%)	<0.01^2^
Myalgia	34(37%)	26(38%)	9(36%)	1.0^2^
Dyspnea	32(34%)	12(18%)	20(80%)	**<0.01^2^**
**Comorbidities**	32(34%)	12(18%)	16(64%)	**<0.01^2^**
Diabetes	11(12%)	6(9%)	5(20%)	0.158^2^
Hypertension	5(5%)	0	5(20%)	
Cardiovascular disease	4(4%)	0	4(16%)	
Chronic obstructive pulmonary disease	8(9%)	6(9%)	2(8%)	1.0^3^
Malignancy	4(4%)	3(4%)	1(4%)	1.0^3^
**Duration (days)**	21.6±6.8	21.8±5.8	21.2±8.9	0.735^1^
**Treatments**				
Antibiotics	71(76%)	68(100%)	3(12%)	<0.0001^3^
Antiviral treatment	71(76%)	68(100%)	3(12%)	<0.0001^3^
Corticosteroids	21(23%)	0	21(84%)	<0.0001^3^
Intravenous immunoglobin	89(96%)	68(100%)	21(84%)	0.004^3^
High-flow nasal cannula oxygen therapy	24(26%)	1(1.5%)	23(92%)	<0.0001^3^
Non-invasive mechanical ventilation	11(12%)	0	11(44%)	<0.0001^3^
Invasive mechanical ventilation	5(5.4%)	0	5(20%)	<0.0001^3^

**Note**: Data are n (%) or mean ± SD, unless otherwise specified. ^1^Mann-Whitney U test;^ 2^Chi-square test; ^3^Fisher's exact test owing to expected count less than 5 for at least one cell.

**Table 2:** Clinical features of patients with COVID-19 on admission.

Clinical features	All patients (n=93)	Survivors (n=68)	Nonsurvivors (n=25)	p value
White blood cell, G/L	4.7(3.8-5.9)	4.6(3.8-5.8)	5.2(3.9-5.9)	0.201^1^
Lymphocyte, G/L	1.2(0.8-1.5)	1.2(0.9-1.6)	0.8(0.6-1.2)	0.001^1^
Neutrophil, G/L	2.9(2.3-4.1)	2.8(2.2-3.6)	3.8(2.7-5.2)	0.045^1^
Platelet, G/L	169(131-207)	181(147-224)	136(112-173)	0.002^1^
Haemoglobin, G/L	131(120-146)	131(120-143)	133.0(16.8)	0.717^1^
NLR	2.8(1.8-4.4)	2.3(1.6-3.8)	0.2(0.1-0.5)	<0.0001^1^
Lymphocyte percentage	24.7(11.1)	27.3(10.6)	17.8(11.4-22.5)	<0.0001^1^
Neutrophil percentage	67.7(12.4)	64.7(11.6)	74.2(12.0)	0.002^2^
Albumin, g/L	37.8(5.8)	38.8(4.9)	33.4(6.0)	<0.0001^2^
Globulin, g/L	28.7(3.91)	28.2(3.7)	29.9(4.2)	0.108^2^
ALT, U/L	27(18.5-42)	27(16-44)	29(22.5-39.8)	0.514^1^
AST, U/L	29(22.8-40)	26(22-35)	39(31-52)	0.001^1^
LDH, U/L	227(180-339)	204(173-248)	373(151)	<0.0001^1^
Creatine kinase, U/L	82.5(47.5-180)	59.5(40.8-116)	186(124-300)	0.001^1^
Creatinine, μmol/L	72.9(58.5-89.6)	70.7(56.6-86.9)	79.8(64.4-92.8)	0.107^1^
BUN, mmol/L	3.9(3.1-4.9)	3.6(2.8-4.6)	4.7(3.8-5.8)	0.005^1^
Total bilirubin, μmol/L	9.4(8.3-12.5)	9.3(8.0-12.1)	9.6(8.6-14.2)	0.374^1^
Direct bilirubin, μmol/L	3.4(3.0-4.9)	3.4(2.9-4.8)	3.5(3.0-4.9)	0.547^1^
Prothrombin time, s	13.1(12.7-13.8)	13.0(12.4-13.5)	13.7(0.75)	0.019^1^
APTT, s	38.9(36.2-42.3)	38.3(4.4)	44.6(9.5)	0.003^2^
ESR, mm/h	30.0(7.8-61.5)	16(6.5-39)	86.8(33.5)	<0.0001^1^
CRP, mg/L	10.8(5.6-32.0)	7.7(3.9-15.7)	77(44)	<0.0001^1^
D-Dimer, mg/L	0.3(0.2-0.6)	0.3(0.2-0.5)	0.6(0.3-2.1)	0.064^1^
Serum ferritin, μg/L	641(417)	489(381)	810(409)	0.094^2^
hsTNI, ng/L	11.4(5.3-23.8)	7(6.7)	13.2(7.6-70.0)	0.022^1^

**Note**: Data are median (IQR) or mean (SD), unless otherwise specified. ^1^Mann-Whitney U test; ^2^independent sample t test. NLR: neutrophil-to-lymphocyte ratio; ALT: alanine aminotransferase; AST: aspartate aminotransferase; LDH: lactate dehydrogenase; BUN: blood urea nitrogen; APTT: activated partial thromboplastin time; ESR: erythrocyte sedimentation rate; CRP: C-reactive protein; hsTNI: high-sensitive cardiac troponin I.

**Table 3:**
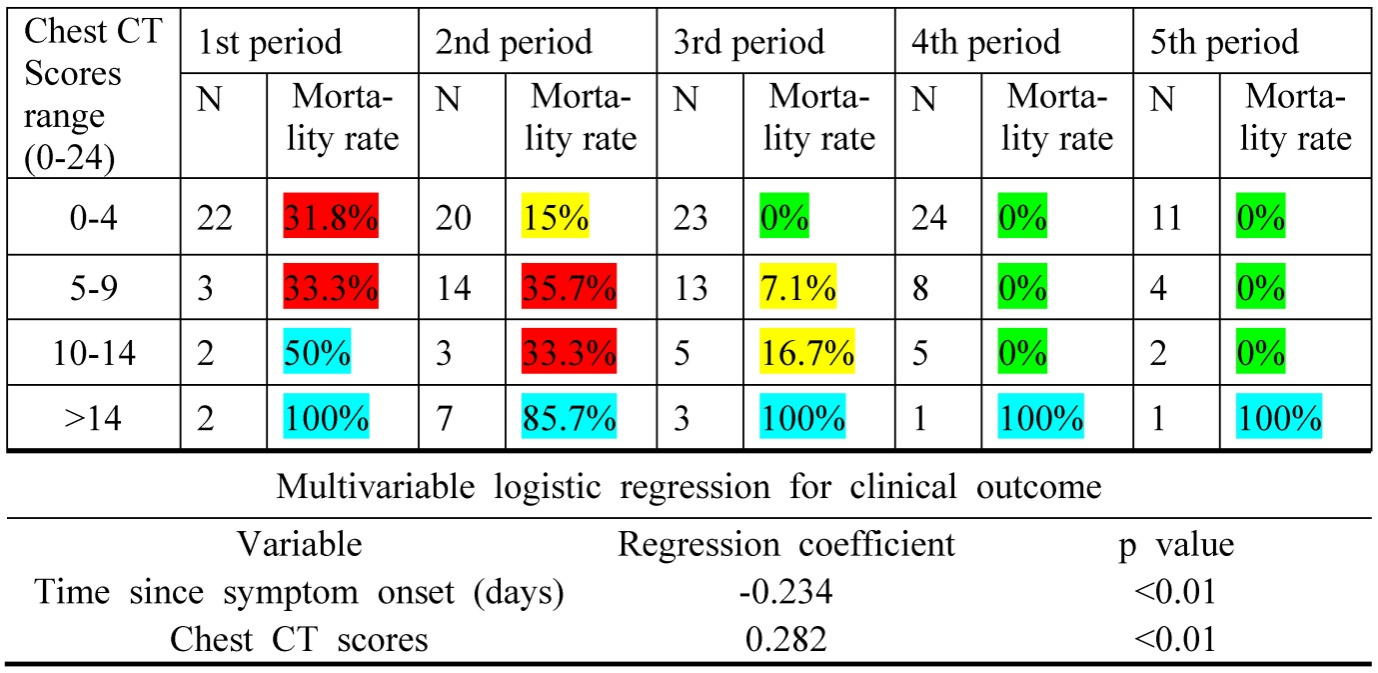
Mortality rate regarded to chest CT scores and time since symptom onset

**Note:** An increasing trend for the mortality rate was observed with the increase in the chest CT scores, as the multivariable logistic regression coefficient for the variable chest CT scores was positive. High chest CT scores in the earlier periods indicated a worse prognosis in the disease than the later ones, as the regression coefficient for the time since symptom onset was negative. N is the number of patients with COVID-19. Green indicates a mortality rate < 5%; yellow, mortality rate between 5% and 30%; red, mortality rate between 30% and 50%; and blue, mortality rate between 50% and 100%.

**Table 4:** Summary of the relationship between chest CT scores and clinical features in COVID-19

Clinical features	p value	The Kendall coefficient
Neutrophil-to-lymphocyte ratio	**<0.01**	0.228
Lymphocyte percentage	**<0.01**	-0.218
Lymphocyte count	**<0.01**	-0.294
Neutrophil percentage	**<0.01**	0.264
D-Dimer	**<0.01**	0.445
Lactate dehydrogenase	**<0.01**	0.477
Erythrocyte sedimentation rate	**<0.01**	0.473

## Abbreviations

COVID-19: coronavirus disease 2019
ARDS: acute respiratory distress syndrome
GGO: ground glass opacity
NLR: neutrophil-to-lymphocyte ratio
LDH: lactate dehydrogenase
ESR: erythrocyte sedimentation rate
CRP: C-reactive protein
ALT: alanine aminotransferase
AST: aspartate aminotransferase
BUN: blood urea nitrogen
APTT: activated partial thromboplastin time
hsTNI: high-sensitive cardiac troponin I
