# Supplementary material for: Association of clinical and radiographic findings with the outcomes of 93 patients with COVID-19 in Wuhan, China
Source: Theranostics. 2020 May 15;10(14):6113–21. doi: 10.7150/thno.46569 (PMC7255034; doi:10.7150/thno.46569)

Supplementary data

I. The definition of the typical CT findings in COVID-19

The typical CT findings for COVID-19 included GGO, crazy-paving pattern, consolidation and mixed pattern. All the demonstrations were defined based on the glossary from the Fleischner Society (Ref. 1).

Mixed pattern is defined as the predominant CT pattern, including ground-glass opacity, reticular, and consolidation (Ref. 2).

Ground-glass opacity (GGO) is defined as hazy increased opacity of lung, with preservation of bronchial and vascular margins on CT scans. It is caused by partial filling of airspaces, interstitial thickening (due to fluid, cells, and/or fibrosis), partial collapse of alveoli, increased capillary blood volume, or a combination of these, the common factor being the partial displacement of air (Ref. 1).

Consolidation is defined as a homogeneous increase in pulmonary parenchymal attenuation that obscures the margins of vessels and airway walls on radiographs and CT scans, and an air bronchogram may be present (Ref. 1).

Crazy-paving pattern is defined as thickened interlobular septa and intralobular lines superimposed on a background of ground-glass opacity, resembling irregularly shaped paving stones. The crazy-paving pattern is often sharply demarcated from more normal lung and may have a geographic outline (Ref. 1).

GGO, consolidation, and crazy-paving pattern are the typical CT features of pulmonary viral pneumonia (Ref. 3, 4), which have also been confirmed for coronavirus disease 2019 (COVID-19) in several previous studies (Ref. 2, 5, 6, 7, 8).

References:

1. Hansell DM, Bankier AA, MacMahon H, McLoud TC, Müller NL, Remy J. Fleischner Society: glossary of terms for thoracic imaging. Radiology. 2008; 246: 697-722.

2. Shi H, Han X, Jiang N, Cao Y, Alwalid O, Gu J, et al. Radiological findings from 81 patients with COVID-19 pneumonia in Wuhan, China: a descriptive study. Lancet Infect Dis. 2020; 20: 425-434.

3. Franquet T. Imaging of pulmonary viral pneumonia. Radiology. 2011; 260: 18-39.

4. Koo HJ, Lim S, Choe J, Choi S-H, Sung H, Do K-H. Radiographic and CT features of viral pneumonia. Radiographics. 2018; 38: 719-739.

5. Bernheim A, Mei X, Huang M, Yang Y, Fayad ZA, Zhang N, et al. Chest CT findings in coronavirus disease-19 (COVID-19): relationship to duration of infection. Radiology, in press. doi: 10.1148/radiol.2020200463.

6. Pan F, Ye T, Sun P, Gui S, Liang B, Li L, et al. Time course of lung changes on chest CT during recovery from 2019 novel coronavirus (COVID-19) pneumonia. Radiology, in press. doi: 10.1148/radiol.2020200370.

7. Chung M, Bernheim A, Mei X, Zhang N, Huang M, Zeng X, et al. CT imaging features of 2019 novel coronavirus (2019-nCoV). Radiology. 2020; 295: 202-207.

8. Wang Y, Dong C, Hu Y, Li C, Ren Q, Zhang X, et al. Temporal changes of CT findings in 90 patients with COVID-19 pneumonia: a longitudinal study. Radiology, in press. doi: 10.1148/radiol.2020200843.

II. Supplementary tables

Table S1. Pulmonary involvement in patients with COVID-19 identified from chest CT.

Pulmonary involvement	All patients (n=93)	Survivors (n=68)	Nonsurvivors (n=25)	p-value
No involvement	4/175(2%)	4/142(3%)	0/33	> 0.05[1]
Unilateral	31/175(18%)	25/142(17%)	6/33(18%)	> 0.05[2]
Bilateral	140/175(80%)	113/142(80%)	27/33(82%)	> 0.05[2]

Note: Data are n (%), unless otherwise specified. [1]Fisher's exact test owing to expected count less than 5 for at least one cell; [2]Chi-square test.

Table S2. The frequency of CT demonstrations in different periods defined based on the time since symptom onset.

Symptoms	All patients (n=93)	Survivors (n=68)	Nonsurvivors (n=25)	p value
**0 - 5 days since symptom onset**				
GGO	26/31(84%)	17/20(85%)	9/12(75%)	> 0.05[1]
Consolidation	16/31(52%)	9/20(45%)	8/12(67%)	> 0.05[1]
Crazy-paving pattern	8/31(26%)	3/20(15%)	5/12(42%)	> 0.05[1]
**6 - 10 days since symptom onset**				
GGO	25/40(63%)	17/28(61%)	10/14(71%)	> 0.05[1]
Consolidation	29/40(73%)	22/28(79%)	8/14(57%)	> 0.05[2]
Crazy-paving pattern	17/40(43%)	9/28(32%)	9/14(64%)	> 0.05[2]
**11 - 15 days since symptom onset**				
GGO	37/46(80%)	33/42(79%)	5/5(100%)	> 0.05[1]
Consolidation	34/46(74%)	30/42(71%)	5/5(100%)	> 0.05[1]
Crazy-paving pattern	12/46(26%)	9/42(21%)	4/5(80%)	**< 0.05[1]**
**16 - 20 days since symptom onset**				
GGO	29/36(81%)	29/35(83%)	0/1(0)	> 0.05[1]
Consolidation	24/36(67%)	23/35(66%)	1/1(100%)	> 0.05[1]
Crazy-paving pattern	1/36(3%)	1/35(3%)	0/1(0)	> 0.05[1]
**>20 days since symptom onset**				
GGO	16/17(94%)	16/17(94%)	1/1(100%)	> 0.05[1]
Consolidation	8/17(47%)	8/17(47%)	1/1(100%)	> 0.05[1]
Crazy-paving pattern	0/17(0)	0/17(0)	0/1(0)	

Note: Data are n (%). [1]Fisher's exact test owing to expected count less than 5 for at least one cell; [2]Chi-square test. GGO: ground glass opacity.

Table S3: Temporal changes in chest CT scores in patients with COVID-19 in each period.

Time since symptom onset (days)	0-5	6-10	11-15	16-20	>20
**Number of chest CT scans**					
survivors	18	29	41	37	17
nonsurvivors	11	15	5	1	1
**Time since symptom onset**					
survivors	3.1(1.8)	9(7-10)	12(11.5-14)	18(16-19)	23(21-24.5)
nonsurvivors	2.5(1.2)	8.4(1.6)	13.4(1.3)	20	26
P value	0.368[2]	0.90[1]	0.35[1]	0.21[1]	0.33[1]
**Chest CT score**					
survivors	2.5(1-4)	4(2.5-6)	4(2-6)	4.7(3.4)	4.6(2.9)
nonsurvivors	2(1-13)	10.9(7.3)	15.8(4.4)	23	20
P value	0.412[1]	**0.006**[1]	**< 0.001**[1]	**< 0.001**[2]	**0.002**[2]

Note: Data are median (IQR) or mean (SD), unless otherwise specified.

[1]Mann-Whitney U test; [2]independent sample t test.

Table S4. Results of linear mixed models with repeated measures.

Clinical features	Within subject test (p value)	Between groups test (p value)	Interaction test (p value)
the chest CT scores	< 0.01	< 0.01	< 0.01
Neutrophil-to-lymphocyte ratio	< 0.01	< 0.01	< 0.01
Lymphocyte percentage	0.071	< 0.01	< 0.01
Lymphocyte count	0.759	< 0.01	< 0.05
White blood cell count	< 0.01	< 0.01	< 0.05
Neutrophil count	< 0.05	< 0.01	< 0.05
Neutrophil percentage	0.163	< 0.01	< 0.01
Platelet count	< 0.01	< 0.01	< 0.05
D-Dimer	< 0.05	< 0.01	< 0.05
Lactate dehydrogenase	< 0.01	< 0.01	< 0.05
Erythrocyte sedimentation	< 0.05	< 0.01	< 0.01

Note: Results of linear mixed models with repeated measures for within subject test over different time periods, between clinical outcome groups test and the interaction test between clinical outcome groups factor and time periods factor.

III. Supplementary figure

Figure S1: The flow chart of patient recruitment.

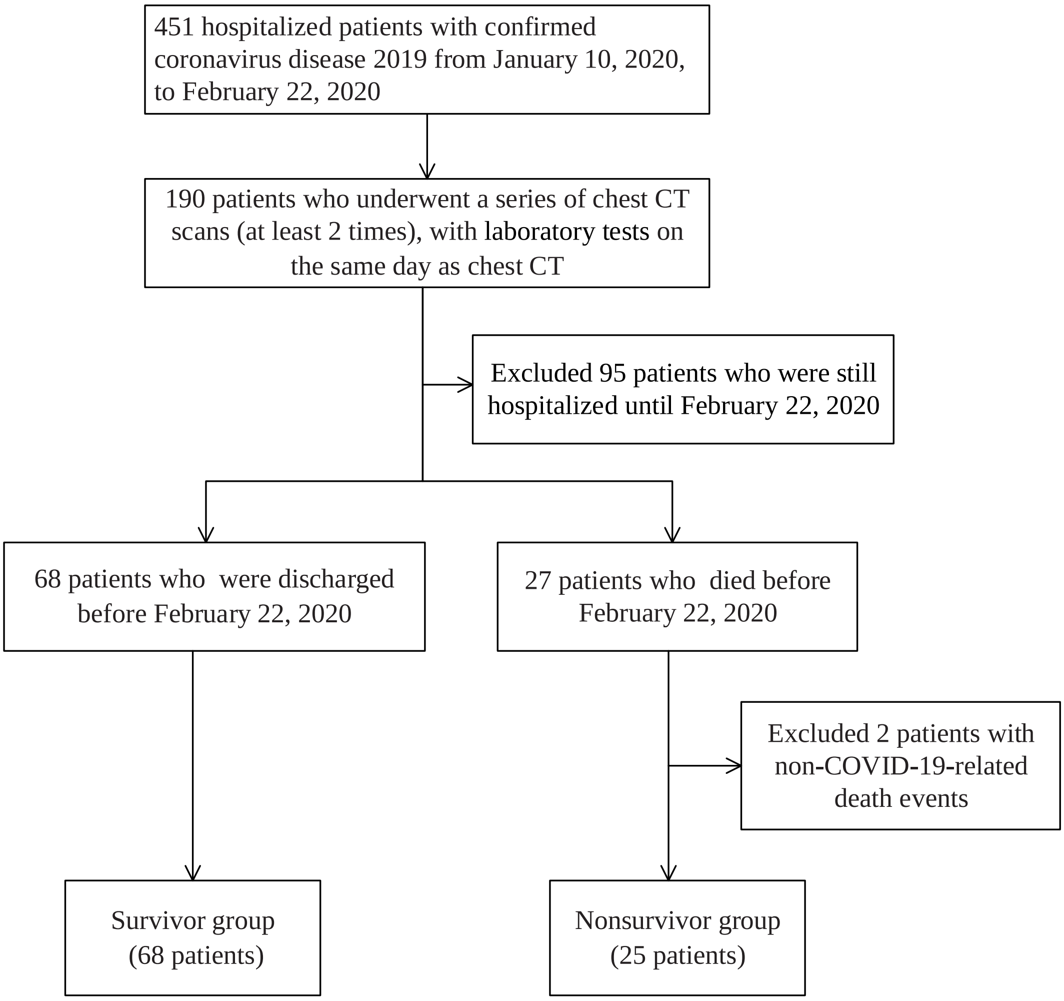

Supplement: Supplementary file 1 — Supplementary figures and tables. [file thnov10p6113s1.pdf]
